# Structure of a DNA‐Stabilized Ag_16_Cl_2_ Nanocluster in Solution

**DOI:** 10.1002/anie.202422432

**Published:** 2025-04-01

**Authors:** Adam F. Sapnik, Giacomo Romolini, Cecilia Cerretani, Tom Vosch, Kirsten M. Ø. Jensen

**Affiliations:** ^1^ Department of Chemistry University of Copenhagen Universitetsparken 5 Copenhagen Ø 2100 Denmark

**Keywords:** Cluster compounds, DNA, Pair distribution function, Silver nanoclusters

## Abstract

We determine the first solution‐state structure of a DNA‐stabilized Ag_16_Cl_2_ nanocluster using X‑ray total scattering and pair distribution function analysis. We find that the structure in solution exhibits both displacive and rotational distortions compared to the known crystal structure. Additionally, our measurements are sensitive toward changes in DNA conformation, revealing that the DNA scaffold in solution exhibits significantly more flexibility than when conformationally locked in the crystalline form. Our results demonstrate the capability to determine the structure of DNA‐stabilized clusters beyond their crystallized form, an essential step toward understanding differences between their solution‐phase and solid‐state photophysical properties.

## Graphical abstract

X‐ray total scattering and pair distribution function analysis are used to determine the first solution‐state structure of a DNA‐stabilized Ag nanocluster.

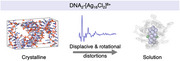

## Introduction

DNA oligomers can template the self‐assembly of atomically precise Ag nanoclusters.^[^ ^1^ ^]^ Since their discovery in 2004,^[^ ^2^ ^]^ the spectroscopic properties of DNA‐stabilized Ag nanoclusters (DNA‐AgNCs) have been studied extensively, often displaying nanosecond‐^[^ ^1^ ^]^ and microsecond‐lived emissions.^[^ ^3^, ^4^, ^5^, ^6^ ^]^ By varying the nucleobase sequence, the number of Ag atoms and, subsequently, the emission wavelength, decay time, quantum yield, and Stokes shift can be varied. However, the rational design of DNA‐AgNCs with specific photophysical properties is challenging due to the very limited examples of well‐characterized atomic structures.^[^ ^7^, ^8^, ^9^, ^10^, ^11^, ^12^ ^]^ This is understandable given their complex structure, which involves the atomic arrangement of the Ag atoms (a mix of neutral and cationic atoms), the coordinate bonds to the DNA, and the overall DNA conformation. It is the combination of all these structural aspects that determines the observed photophysical response.^[^ ^13^, ^14^, ^15^ ^]^

While the one‐pot synthesis is straightforward, involving only aqueous solutions of DNA, silver nitrate, and a reducing agent such as sodium borohydride, thorough purification is vital to ensure the isolation of the desired atomically precise DNA‐AgNC.^[^ ^1^ ^]^ Techniques like size exclusion and high‐performance liquid chromatography remove unwanted Ag nanoparticles, free DNA (likely complexed with Ag cations), and other unwanted DNA‐AgNCs, facilitating detailed photophysical characterization, mass spectrometry measurements, and potential crystallization. An example of such purified DNA‐AgNC is DNA_2_‐[Ag_16_Cl_2_]^8+^, where two DNA decamers (5′‐CACCTAGCGA‐3′) and two Cl^–^ stabilize an Ag_16_ nanocluster. DNA_2_‐[Ag_16_Cl_2_]^8+^ displays near‐infrared emission and an unusually large Stokes shift.^[^ ^13^, ^16^ ^]^

Currently, the structural characterization of DNA‐AgNCs has relied on single‐crystal X‐ray diffraction (SCXRD) methods, which were first demonstrated in 2019.^[^ ^7^, ^8^ ^]^ While only limited examples exist, they have enabled promising chemical linking strategies (e.g., peptides and proteins clicked to DNA‐AgNC)^[^ ^17^ ^]^ and electronic structure calculations.^[^ ^18^, ^19^ ^]^ However, several challenges have been faced in the SCXRD studies of DNA‐AgNCs. For example, difficulties in resolving the DNA conformation have been reported recently for DNA_2_‐[Ag_11_]^7+^, where only an approximate outline of the Ag atoms’ positions was obtained.^[^ ^12^ ^]^ In the case of DNA_2_‐[Ag_11_]^7+^, many modifications to the DNA sequence were necessary to achieve successful crystallization and well‐diffracting crystals.^[^ ^12^ ^]^ Another crucial consideration is that the atomic structure of the crystallized phase is not necessarily the same as that of the solution phase. For DNA_2_‐[Ag_16_Cl_2_]^8+^, for example, a blue shift of ≈30 nm in the emission spectrum was observed in the crystalline state compared to in solution, suggesting that some differences in atomic structure are present.^[^ ^8^, ^9^, ^10^, ^11^ ^]^ Aside from the aforementioned examples, no other crystal structures have been reported, and the vast majority of DNA‐AgNC studies have been carried out in the solution phase.

Mass spectrometry,^[^ ^20^, ^21^ ^]^ circular dichroism,^[^ ^22^ ^]^ infrared spectroscopy,^[^ ^23^, ^24^ ^]^ and X‐ray spectroscopy^[^ ^25^ ^]^ have been applied to characterize DNA‐AgNCs in solution. While they offer valuable insights into the overall understanding of DNA‐AgNCs, they lack the detailed atomic‐level resolution required to fully exploit the structure–property relationships of DNA‐AgNCs. Nuclear magnetic resonance spectroscopy has the potential to provide structural insights, but so far, only ^35^Cl‐NMR has been performed to evaluate the position of the Cl^–^ ligands of DNA_2_‐[Ag_16_Cl_2_]^8+^ in solution.^[^ ^22^ ^]^

X‐ray total scattering and pair distribution function (PDF) analysis offer potential to probe the structure of AgNCs in solution. Avoiding the necessity for crystallization is a great advantage of this technique and allows for structure–property relationships to be established for the solution phase. With total scattering methods, the diffuse scattering arising from matter with no long‐range order can be analyzed to yield structural models. The Fourier transform of the total scattering yields the PDF, an intuitive mapping of the pairwise atomic correlations within a structure.^[^ ^26^, ^27^ ^]^ PDF methods have previously been applied for structural studies of small cluster structures.^[^ ^28^, ^29^, ^30^, ^31^, ^32^, ^33^ ^]^

In this study, we perform X‐ray total scattering measurements and PDF analysis to determine the solution‐state structure of DNA_2_‐[Ag_16_Cl_2_]^8+^. Structural refinements show that measurable differences from the crystallized form can be observed, exhibiting displacive and rotational distortions alongside increased flexibility in the DNA scaffold.

## Results and Discussion

### X‐Ray Total Scattering Measurements

**Figure 1:**
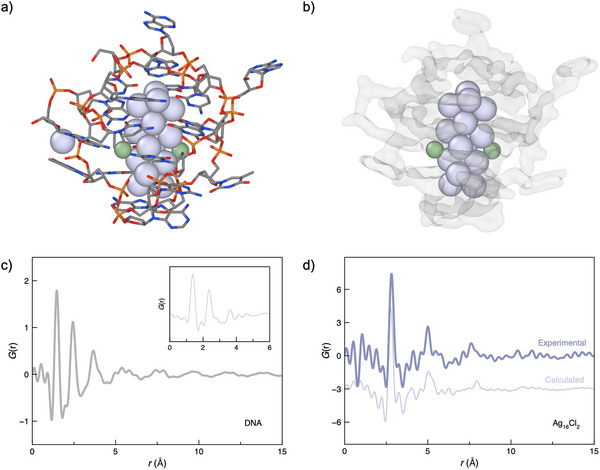
Atomistic X‐ray single crystal structure of DNA_2_‐[Ag_16_Cl_2_]^8+^ where DNA is represented as a) a stick model and b) a semi‐transparent surface mesh. Ag (purple), Cl^–^ (green). c) Experimental PDF of aqueous DNA, the inset shows a linear combination of PDFs calculated from the nucleobase molecules combined in the ratio present in the DNA strand. d) Experimental PDF of DNA_2_‐[Ag_16_Cl_2_]^8+^ in solution after background subtraction using aqueous DNA (dark purple) and calculated PDF from the Ag_16_Cl_2_ nanocluster extracted from (a), PDFs offset for clarity.

We began by measuring the X‐ray total scattering from the DNA oligomers in aqueous solution at concentrations similar (≈12.5 mm) to those used for the DNA_2_‐[Ag_16_Cl_2_]^8+^ sample. The PDF for DNA contains two sharp peaks (1.46 and 2.43 Å), followed by a broader peak (3.69 Å), two low‐intensity regions centerd around 5.55 and 7.55 Å, and was featureless beyond 15 Å (Figure 1c). These observations are typical for organic polymers, where sharp low‐*r* peaks arise from the well‐defined monomer local structure and broader regions corresponding to the less well‐defined molecular packing. Specifically, the peaks at 1.46 and 2.43 Å correspond to the C–C/P–O and O–O distances, respectively. The peak at 3.69 Å arises from further nucleobase correlations and hence becomes broader, given the increased number of pairwise correlations. A linear combination of the PDFs calculated from the nucleobases combined in the correct ratio reproduces the local structure observed in the experimental PDF well (Figure 1c inset; Figure S1). Finally, the two broad regions in the PDF arise from the secondary structure of the oligomers, which is largely ill‐defined due to the molecular tumbling occurring in the solution.

Having demonstrated the ability to detect aqueous DNA in PDF analysis, we then measured the total scattering from DNA_2_‐[Ag_16_Cl_2_]^8+^ in solution (≈10 mm, based on the DNA concentration). By carefully subtracting the aqueous DNA signal from the DNA_2_‐[Ag_16_Cl_2_]^8+^ data, we could minimize the contributions from the local structure of the nucleobases to isolate the correlations from the Ag_16_Cl_2_ core (Figures S2 and S3). Quite remarkably, we could detect a clear signal from the Ag_16_Cl_2_ core in the solution. The PDF contains a strong peak at 2.80 Å corresponding to the first Ag–Ag distance, another intense peak at 5.00 Å, and two broad oscillations in the baseline between 5 and 10 Å. Beyond this, the PDF was largely featureless. The position of the Ag–Ag peak is intermediate to that of diatomic (2.53 Å) and bulk (2.89 Å) Ag.^[^ ^34^, ^35^ ^]^ However, theoretical studies on AgNC have suggested that the evolution of this distance between the diatomic and bulk is not necessarily continuous as cluster size increases due to a combination of structural transitions, electronic effects, and variations in the coordination environment.^[^ ^35^ ^]^ Meanwhile, the termination of peaks in the PDF provides an approximate measure of the structural coherence length in the cluster (≈10–12 Å), which is consistent with the size of the Ag_16_Cl_2_ core. Furthermore, given that the PDF represents a time‐averaged snapshot of the structure, the featureless high‐*r* region suggests the clusters are highly dynamic in solution with little spatial coherence between clusters, at least on the timescales measured here. Both DNA and DNA_2_‐[Ag_16_Cl_2_]^8+^ were evaluated to be stable with respect to the X‐ray beam, displaying negligible changes throughout the measurement (Figure S4).

Given that the atomic structure of the DNA_2_‐[Ag_16_Cl_2_]^8+^ from SCXRD was previously reported by Cerretani et al., we extracted the atomic coordinates of the Ag_16_Cl_2_ core and calculated the PDF.^[^ ^8^ ^]^ Note that the two atoms with low electron density, initially identified as Ag, were revealed by later studies to be Cl^–^.^[^ ^16^ ^]^ Visual comparison to our experimental data shows appreciable similarity (Figure 1d). As such, we were confident that the detection of such small clusters is possible in solution. However, closer inspection reveals slight discrepancies in the PDFs, suggesting subtle yet measurable differences in the structure of the Ag_16_Cl_2_ core—differences that may account for the observed variations in emission spectra in solution when compared to the crystalline state.

### Structural Modeling of the Nanocluster

**Figure 2:**
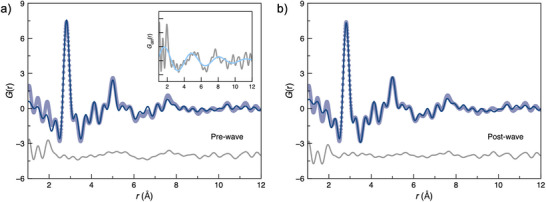
a) Experimental data (purple circles), refined data (blue) and difference (grey) Ag_16_Cl_2_ refinement where atomic positions are refined, inset shows the difference curve and the refinement against the wave function and b) refinement after including the empirical wave function.

Subsequently, the atomic structure of the extracted Ag_16_Cl_2_ core was refined against the experimental PDF. This was principally achieved by refining the position of each atom (see Supporting Information). The refined PDF exhibits a noticeably better agreement with the experimental data than the as‐reported single‐crystal Ag_16_Cl_2_ structure (Figure 2a). This is also reflected in a reduction in *R*_w_ from 0.48 to 0.25 before and after refinement, respectively. We acknowledge the potential for overfitting the PDF data at this stage and address this in later analysis.

The difference function from this initial refinement has a very characteristic sinusoidal shape, suggesting some structural coherence that is unaccounted for by the model (Figure 2a inset). Residual curves such as this have previously been reported to arise from solvent‐ordering effects induced by colloidal nanoparticles or ionic clusters in solution.^[^ ^29^, ^36^ ^]^ Zobel et al. have proposed an empirical model for these solvation effects in the form of an exponentially damped sine wave.^[^ ^36^ ^]^ Introducing this function to our refinement gives us a strikingly good agreement between the model and experimental data (Figure 2b). The physical interpretation of this residual function will be discussed later. For now, we comment that this wave function captures our system's “solvation‐like” environment and return to this discussion below.

One of the most significant challenges in structure determination from PDF data is uniqueness.^[^ ^37^, ^38^ ^]^ A fundamental limitation is a common lack of information contained within the PDF with respect to the number of parameters in the model, so it is often unrealistic to expect a unique structural refinement.^[^ ^39^, ^40^ ^]^ This is true for solids but even more so for structures in solution where atomic motion will inherently be less restricted. In this context, we did not necessarily expect a unique refinement, given the structure in solution is likely to be dynamic to some degree. Indeed, this was true and led us to evaluate our refinement process in detail.

In our experience, three outcomes are typically observed when performing cluster refinements when the information content is somewhat limited. First, the refinement simply does not converge. Second, the refinement converges, but the structure is no longer physically sensible. Third, as is the case here, the refinement converges, but the parameters are correlated. In testing the refinement process, we found that perturbations in the starting model subsequently led to slight differences in the refined structure. While these variations were found to be reproducible (i.e., there was a one‐to‐one mapping between initial structure and refined structure), they nonetheless suggested issues with uniqueness. To overcome this and ensure a meaningful structure refinement of the Ag_16_Cl_2_ core, we developed a statistical approach toward the refinement.

First, the structure of the Ag_16_Cl_2_ core (our starting model) was perturbed by randomly displacing each atom by up to 0.2 Å along each direction. This was performed 500 times (guided approximately by the number of unique refinement parameters (55) multiplied by 10) to generate a library of initial Ag_16_Cl_2_ core models. The model generation is described in detail in the Supporting Information. Each model was refined as described above, refining the atomic positions and the empirical wave function. The refinements successfully converged, achieving fits comparable to that in Figure 2b, with *R*_w_ values in the range of 0.118 to 0.187 and a mean value of 0.179 (Figure S5). Thus, we obtain 500 refined structural models for the Ag_16_Cl_2_ core. Our approach is inspired by reverse Monte Carlo methods, where a large supercell is typically refined against the experimental data to sample all possible structural variations permitted by the data.^[^ ^41^ ^]^

**Figure 3:**
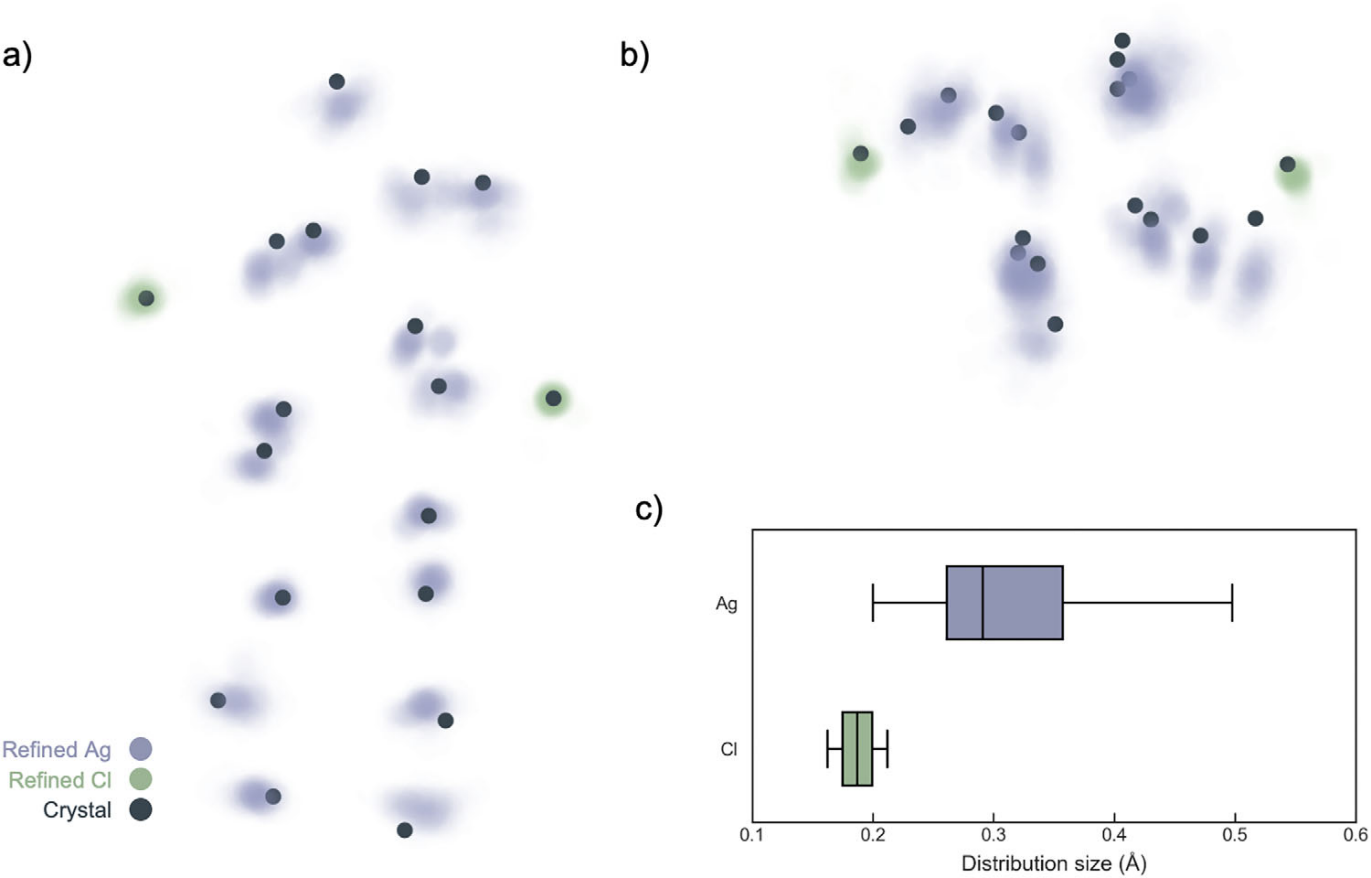
Representation of the refined structures (semi‐transparent points) and the original crystal structure (opaque points) viewed a) perpendicular and b) along the central axis of the cluster. c) Box plot representing the variation in the distribution of atomic positions. The minimum, maximum, median, and interquartile range of the distribution size were used to construct the box plot. Ag (purple) and Cl^–^ (green).

The ensemble of structures was then visualized to assess variations across the refinements (Figure 3a,b). The atomic positions from each refinement are shown as semi‐transparent points; thus, darker regions of the figures correspond to positions with higher likelihood. For comparison, the original positions from the crystal structure are shown as opaque points.^[^ ^8^ ^]^ We found that the distribution of the Cl^−^ positions was quite narrow, as shown in Figure 3c, and exhibited only a slight variation from the crystal structure. Meanwhile, the Ag positions exhibited much broader distributions, with those toward the ends of the cluster exhibiting more significant variations. As the Cl^–^ are coordinatively bound to two Ag, they are effectively anchored in position, hence their limited displacement. Conversely, the DNA experiences more dynamic fluctuations in solution than in the crystal phase, allowing the Ag to rattle around within the DNA scaffold.^[^ ^16^ ^]^ Most importantly, we find that the positions of the reported Ag_16_Cl_2_ crystal structure lie almost exclusively outside of the refined distribution for all atoms. Crucially, this demonstrates a statistically significant difference between the known crystalline structure of Ag_16_Cl_2_ and the structure in solution.

### Nanocluster Model Evaluation

**Figure 4:**
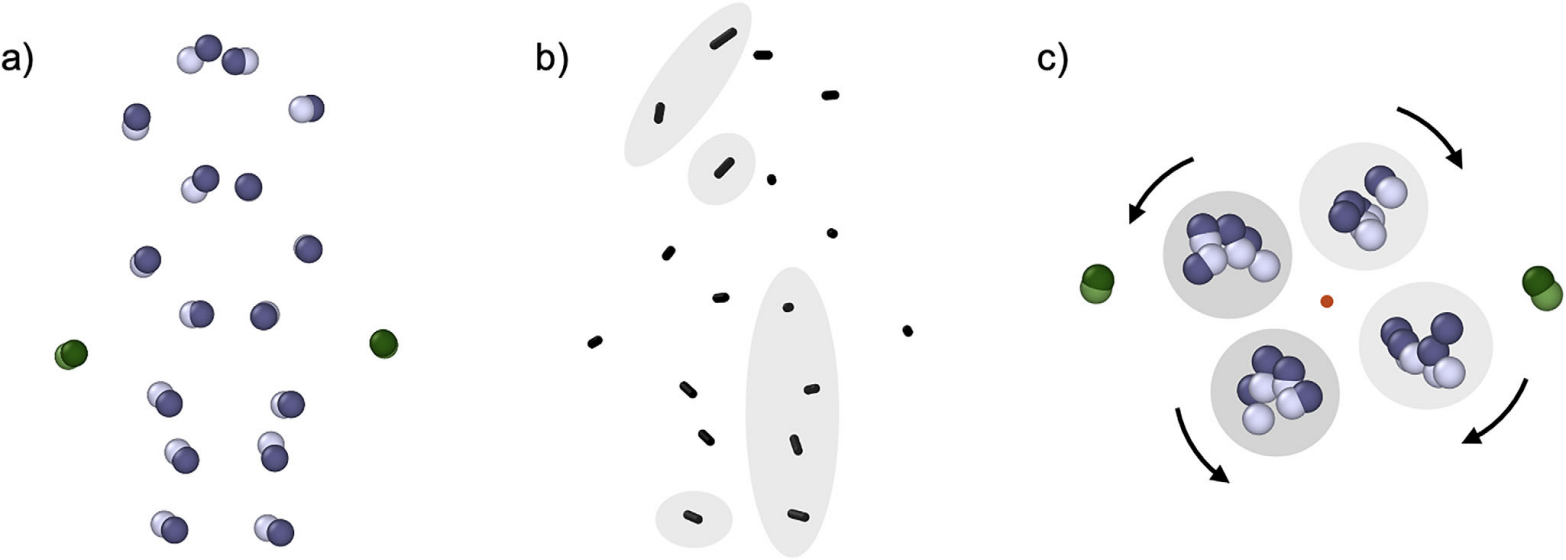
a) The original crystal (dark) and refined average (light) structures of the Ag_16_Cl_2_ core (Ag purple and Cl^−^ green), b) displacement vectors between the original and refined positions, vectors with a magnitude greater than 0.25 Å are shaded in grey and the cluster is in the same orientation as in (a); and c) view along the principal axis (orange dot) of the cluster highlighting the rotational nature of the displacements. Groups of atoms displaying clockwise and counterclockwise rotations are highlighted in light and dark grey, respectively.

The ensemble of refined atomic positions was then collapsed into a single average structure to facilitate further geometric comparisons with the reported crystal structure (Figure 4a). By computing the displacement vector between the initial crystal structure and the refined average position for each atom, two observations were made regarding the spatial variation of the displacement magnitude. This is illustrated in Figure 4b, where the vectors between the initial and refined positions are visualized. First, a spatial dependence of the displacement magnitude was noted; Cl^−^ displayed minimal displacement from the crystal structure, while Ag exhibited larger displacements. Ag toward the middle of the cluster showed smaller magnitudes of displacement compared to those at the ends of the cluster. The magnitude of the displacement varied between 0.20 and 0.71 Å, with a mean value of 0.38 Å.

Second, it was observed that the direction of the displacement vector was not random; there appeared to be some degree of rotation of the vectors along the axis of the cluster (Figure 4c). To quantify this rotational behavior, principal component analysis was performed, and all atoms were projected into the plane perpendicular to the principal axis of the cluster (see Supporting Information). The degree of rotation between the initial and average refined position about the principal axis was then computed (Figure S6). These values varied from −8.77° to 20.12° with a mean of 3.53°.

Upon further inspection, we found that the directions of these rotations were highly correlated. When the Ag_16_Cl_2_ nanocluster is viewed along the principal axis, four channels of Ag atoms can be observed, as seen in Figure 4c. Two of these channels exhibit a clockwise rotation about the principal axis, while the other two show counterclockwise behavior. The mean values for the rotation of the four separate channels were 8.61° and 12.23° for the clockwise rotations and −3.51° and −3.22° in the counterclockwise direction. The (counter)clockwise direction of the rotation correlates with which of the two DNA strands the Ag is coordinated by. For clockwise rotation, seven out of eight of the Ag are coordinated either entirely or partially by the same DNA strand (Figure S7). The same is observed for the counterclockwise rotation, seven out of eight Ag are coordinated either entirely or partially by the other DNA strand.

### Insight into DNA Structure

Given the importance of the DNA environment in energetically stabilizing the Ag_16_Cl_2_ cluster and our sensitivity toward DNA in aqueous solution, we sought to investigate the conformational flexibility of DNA around the Ag_16_Cl_2_ core in solution, in particular, how it compares to free single‐stranded DNA in aqueous solution and the DNA wrapped around Ag_16_Cl_2_ in the crystal structure. As their name implies, DNA‐stabilized Ag nanoclusters have an intimate relationship between the Ag core and DNA. Specifically, in the crystallized DNA_2_‐[Ag_16_Cl_2_]^8+^, the strands tightly encase the Ag_16_Cl_2_ core such that it is almost entirely occluded from the surrounding solvent. Intuitively, we anticipate the DNA is more dynamic in solution due to the molecular tumbling and interactions with the solvent, which, in turn, gives rise to the distorted Ag_16_Cl_2_ core. To evaluate this hypothesis, PDFs simulated from the DNA structure in the reported DNA_2_‐[Ag_16_Cl_2_]^8+^ crystal structure were compared to free DNA in aqueous solution and DNA around the Ag_16_Cl_2_ core in solution. For simplicity, we denote these three forms as DNA (crystal), DNA (aqueous), and DNA (cluster), respectively.

**Figure 5:**
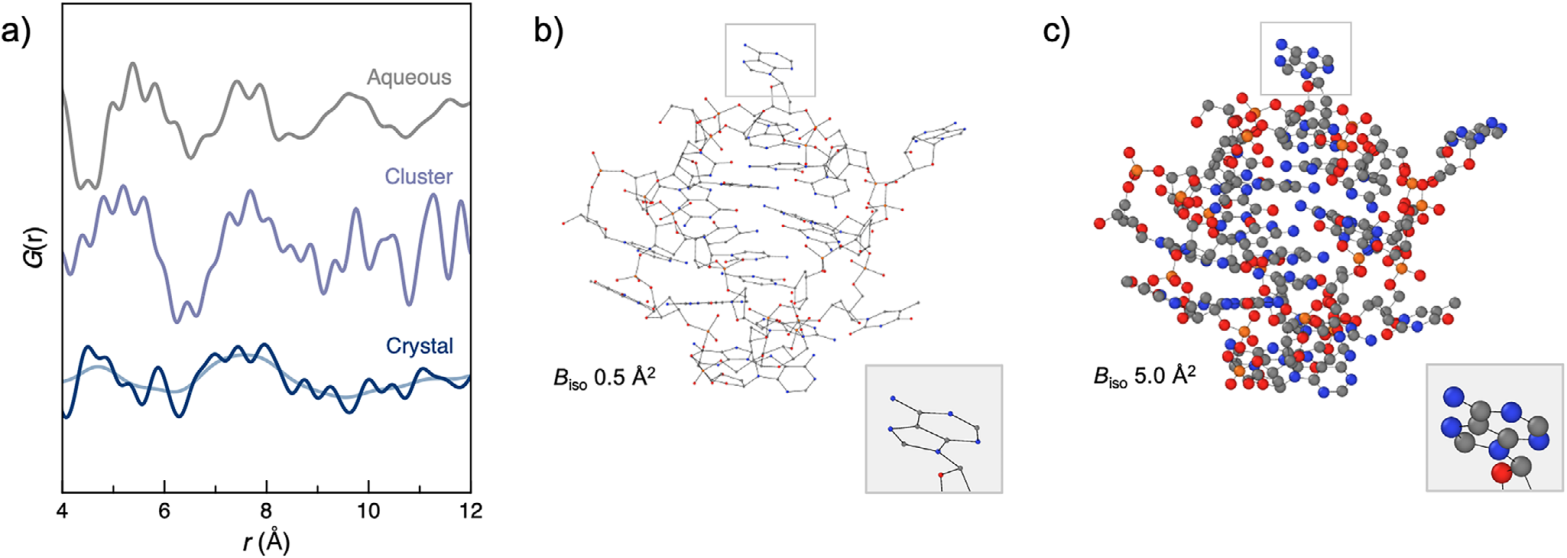
a) Comparison between free DNA in solution (gray), DNA around the cluster in solution (purple), and DNA extracted from the crystal structure calculated with *B*_iso_ values of 0.5 Å^2^ (dark blue) and 5.0 Å^2^ (light blue). Thermal ellipsoid diagrams of the extracted DNA structure corresponding to *B*_iso_ values of b) 0.5 Å^2^ and c) 5.0 Å^2^ represented at 90%. For visual clarity, the inset highlights an arbitrary region of the structure.

As discussed earlier, the PDF for DNA (aqueous) was measured directly and exhibited sharp peaks from the local structure of the nucleobases and additional broader features along the chain of the DNA strand (Figure 5a). Beyond this, the medium‐range order is not well‐defined due to the random conformation of the chain in solution. DNA (aqueous) is not strongly bound in a particular conformation; hence, its PDF serves as a baseline for our interpretation, representing the least‐ordered form of “free” DNA.

We can access the signature of DNA (cluster) via the difference function from the fit in Figure 2a. In producing the PDF presented in Figure 1d, the signal for DNA (aqueous) was subtracted as the background contribution before the Fourier transform to obtain the PDF (Figure S3). While this successfully removed the contributions from the DNA's local structure from the PDF, should there be any differences in the medium‐range structure of DNA (cluster) compared to DNA (aqueous), then this contribution will still be present in the PDF. We would not expect DNA (cluster) to possess a random conformation, given that it is wrapped around the Ag_16_Cl_2_ core and bound via coordinate bonds. Hence, this should contribute an additional structured signal to the experimental PDF compared to DNA (aqueous). After the first stage of our modeling strategy, where the structure of the Ag_16_Cl_2_ core is refined, the extra structured signal remains unaccounted for and is, therefore, present in the residual curve. We can, thus, extract the average difference function from the 500 refinements (before including the empirical wave function) to obtain the PDF for DNA (cluster). This function is plotted in Figure 5a. This PDF contains two broad features centered around ≈5.2 and 7.7 Å. We focused on these two features in the PDF and were cautious to avoid over‐interpretation of the higher‐*r* features, which are likely due to noise.

To determine the PDF of DNA (crystal) for comparison, we used the reported single‐crystal structure model for DNA_2_‐[Ag_16_Cl_2_]^8+^ and extracted the atomic positions of the DNA. The PDF was first calculated using an isotropic displacement parameter (*B*_iso_) of 0.5 Å^2^ as anticipated for a crystalline solid. As expected, the PDF has relatively narrow peaks (Figure 5a). However, the sheer number of atomic pairs quickly leads to overlapping features. To mimic the higher mobility of the DNA while in solution, the PDF was then calculated using a larger *B*_iso_ value of 5 Å^2^. This has the effect of broadening the peak widths, particularly at higher‐*r*, which, in turn, results in greater resemblance to DNA around the cluster in solution. Using a simple statistical model, we were able to identify a *B*_iso_ value of 2.0 Å^2^ as an optimum *B*_iso_ parameter for describing DNA (cluster) in terms of DNA (crystal) (Figure S8). This *B*_iso_ value of 2.0 Å^2^ equates to a mean instantaneous atomic displacement of 0.16 Å, giving us an approximate measure of the degree of motion in solution. It is this flexibility in DNA (cluster) that allows for the distribution of atomic positions within the Ag_16_Cl_2_ core to exist, as the atoms are not as tightly constrained as in the crystalline state. Nonetheless, even in this more flexible state, some degree of interaction between the DNA and Ag_16_Cl_2_ core is maintained, leading to the observed non‐random distortions.

From these investigations and the comparisons shown in Figure 5a, we can make several inferences about the DNA structure in DNA_2_‐[Ag_16_Cl_2_]^8+^ in solution. First and foremost, we can conclude that DNA (cluster) is not identical to DNA (aqueous) or DNA (crystal); however, several similarities are observed. Qualitatively, DNA (cluster) is most similar to DNA (aqueous) from the overall appearance of the PDF. The similarity toward DNA (crystal) only becomes apparent when the atomic displacement parameter is large enough to broaden the PDF significantly and when the *x*‐axis is offset to account for swelling (additional water) present in the solution state versus the crystalline state. Ultimately, we arrive at the same conclusion whether we interpret the DNA (cluster) as free DNA or highly disordered crystalline DNA. That is, there is a significant degree of “floppiness” in the DNA chains around Ag_16_Cl_2_ in solution. If we were to speculate, these results may point toward a degree of dynamics in the structure, especially on the timescales studied here.

## Conclusion

We have demonstrated the ability to detect DNA‐AgNCs in solution for the first time. Using this, we have determined the core atomic structure of DNA_2_‐[Ag_16_Cl_2_]^8+^. Most notably, we find that the structure in solution has measurable differences from that reported from single‐crystal studies and is characterized by displacive and rotational distortions. There was a spatial dependence in both the magnitude and direction of the atomic displacements between the crystal and solution structure. Ag toward the ends of the cluster exhibited larger displacements, while Cl^–^ displayed the smallest variations. The displacement of Ag was not random; rather, it involved a correlated rotation around the principal axis. The direction of this rotation was dependent on which DNA strand the Ag was coordinated by. Our measurements also demonstrated sensitivity toward the conformational structure of DNA, and analysis revealed a dynamic structure of the DNA in solution compared to the crystal. Although these distortions do not render the structure of DNA_2_‐[Ag_16_Cl_2_]^8+^ completely unrecognizable in solution, they may be substantial enough to account for the differences observed in emission spectra between the solid and solution states. In other systems, even greater differences in photophysical properties have been reported between these states. In such cases, more significant variations in atomic structure are likely. For example, in (DNA)_2_‐[Ag_11_]^7+^, an additional emission band with long‐lived character was observed only in the solid state.^[^ ^6^ ^]^ Establishing the differences in atomic structure for this system would enable us to identify structural features that give rise to this extra emission band; such information could prove crucial for designing time‐gated imaging applications. By enabling atomic structural characterization in solution, we have bridged the gap between current solid‐state structural characterization and photophysical properties observed in solution. This, in turn, will allow for the rational design of DNA‐AgNC systems for applications in bio‐imaging and sensing.

## Author Contributions

A.F.S., G.R., K.M.Ø.J., and T.V. conceptualized the project. G.R. and C.C. prepared the samples. A.F.S. performed the X‑ray total scattering measurements, data analysis, and structural modeling and wrote the manuscript. All authors contributed to the final version. T.V. and K.M.Ø.J. acquired funding and resources.

## Conflict of Interests

The authors declare no conflict of interest.

## Supporting information



Supporting Information

## Data Availability

The data that support the findings of this study are available from DOI: 10.5281/zenodo.15069353.
