# Supplementary material for: Structure of a DNA‐Stabilized Ag16Cl2 Nanocluster in Solution
Source: Angew Chem Int Ed Engl. 2025 Apr 1;64(22):e202422432. doi: 10.1002/anie.202422432 (PMC12105681; doi:10.1002/anie.202422432)
Supplement: Supplementary file 1 — Supporting Information [file ANIE-64-e202422432-s001.pdf]

Structure of a DNA-Stabilized $Ag_{16}Cl_2$ Nanocluster in Solution

SUPPLEMENTARY INFORMATION

Adam F. Sapnik,[a]* *Giacomo Romolini,*[a] *Cecilia Cerretani,*[a]
Tom Vosch[a]* *and Kirsten M. Ø. Jensen.*[a]*

a. Department of Chemistry, University of Copenhagen,
Universitetsparken 5,2100 Copenhagen Ø, Denmark.

Email: afs@chem.ku.dk, tom@chem.ku.dk and kirsten@chem.ku.dk

Table of Contents

EXPERIMENTAL METHODS 3

SYNTHESIS 3

X-RAY TOTAL SCATTERING 3

PDF MODELLING 3

SUPPLEMENTARY DATA 4

APPENDIX A 10

REFERENCES 11

Experimental Methods

Synthesis

Due to the large sample volume required for PDF measurements, two DNA-AgNCs were mixed together after preparing them separately. The two clusters are exactly the same, with the only difference given by the replacement in the DNA sequence of two guanosines with the corresponding artificial analogues (inosine), which lacks the amino group bound to C2. The two DNA_2-$[Ag_{16}Cl_2]^{8+}$ clusters were synthesised following the procedure reported by Bogh *et al.*[1] Briefly, an aqueous solution of DNA (5'-CACCTAXCXA-3' where X = G or inosine) was combined with silver nitrate in a 10 mM ammonium acetate (NH_4OAc) solution at pH 7.0 before being reduced by sodium borohydride. The final ratio between the three components [DNA]:[$AgNO_3$]:[$NaBH_4$] was 25 μM:187.5 μM:93.75 μM. The sample was then stored in the fridge for three days before HPLC purification, after which the solvent was exchanged for a 10 mM NH_4OAc solution. HPLC methods and chromatograms are reported in References 1 and 2. Spectroscopic measurements were then performed to confirm that a pure sample was obtained. As mentioned above, the AgNC templated by the inosine mutant of the DNA was previously demonstrated to possess a very similar crystal structure, and the calculated PDFs were almost identical [**Appendix A**].[2,3]

X-ray total scattering

X-ray total scattering data were measured using the DanMax beamline (proposal ID 20231682) at MAX IV (Sweden). The sample was loaded into a 1.0 mm Kapton capillary and sealed with epoxy glue. In addition, an empty capillary and capillaries loaded with water, aqueous ammonium acetate (100 mM) and aqueous DNA (12.5 mM) were also measured to facilitate accurate background subtraction. Data were measured in the range ~ 0.5 $Å^{-1} < Q < 17.5$ $Å^{-1}$ at room temperature for five minutes using an energy of 35 keV and a DECTRIS PILATUS3 X CdTe 2M area detector. Each five-minute measurement comprised 300 one-second exposures, subsequently averaged before further processing. Total scattering data reduction was carried out using PDFGetX3.[4] The Fourier transform was performed in the interval 2 $Å^{-1} < Q < 15.0$ $Å^{-1}$ using a $Q_{\text{max inst}}$ of 17.5 $Å^{-1}$ and an r_{poly} of 1.0.

PDF modelling

Analysis of the PDFs was performed using DiffPy-CMI.[5] The single crystal structure reported in Ref. 3 was used as the starting model. From this CIF, both the $Ag_{16}Cl_2$ nanocluster and DNA structure were extracted. Note that the two atoms with low electron density, identified initially as silvers, were demonstrated to be chlorides by Gonzàlez-Rosell *et al.*[6] Cluster refinements were performed using the Debye scattering equation, and the positions were allowed to vary by up to 1 Å in each direction. The residual was then fit using the empirical damped sinusoidal function from Zobel *et al.* in Ref. 7. The library of 500 starting configurations was generated by randomly displacing the positions of each atom in the $Ag_{16}Cl_2$ cluster with an upper limit of 0.2 Å along each direction. Preliminary investigations showed that successful refinement could not be achieved by (i) refinement of only Cl positions or (ii) refinement of B_{iso} while maintaining the original crystal structure atomic positions. Refinement of only the Ag positions produced a marginally worse fit than the final refinement approach. The final refinement strategy involved iteratively refining the $Ag_{16}Cl_2$ cluster's atomic structure alongside the empirical wave function from Ref. 7, such that $G(r)_{model} = G(r)_{Ag_{16}Cl_2} + G(r)_{wave}$. This ensures that the atomic structure of $Ag_{16}Cl_2$ does not refine in such a way as to compensate for the additional structuring of the DNA signal. The direction of the displacement vector between the initial and refined positions was visualised as a vector map in **Figure 4b** of the main text. The direction of these vectors did not appear random, and visual inspection suggested that some atoms rotate in the same direction about the long axis of the cluster. Hence, we sought to capture this degree of rotation by quantifying the angle formed between the initial and final position about the long axis of the cluster. For this rotational analysis, first, the principal axis of the $Ag_{16}Cl_2$ cluster was determined using principal component analysis as implemented in scikit-learn.[8] All atoms were then projected onto a plane orthogonal to the principal axis. The degree of rotation was then evaluated from the before and after coordinates about the principal axis.

Supplementary Data

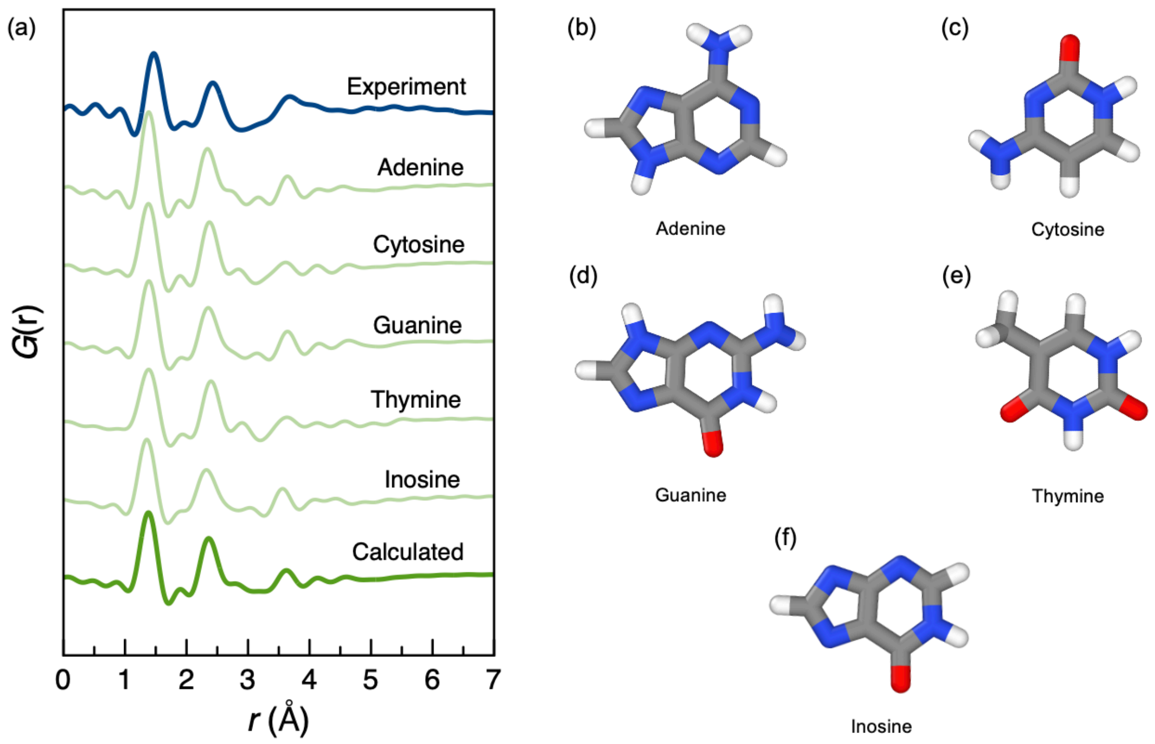

Figure S1 (a) Comparison between the experimental PDF for aqueous DNA, calculated PDFs from the four nucleobases and a linear combination of the four bases in the CACCTAXCXA composition. We note that the first peak of the experimental PDF is slightly broadened due to contributions from the P–O bond of the phosphate backbone, which occurs at 1.59 Å. **(b to f)** Atomic structures of the five nucleobases. C (grey), N (blue), O (red) and H (white).

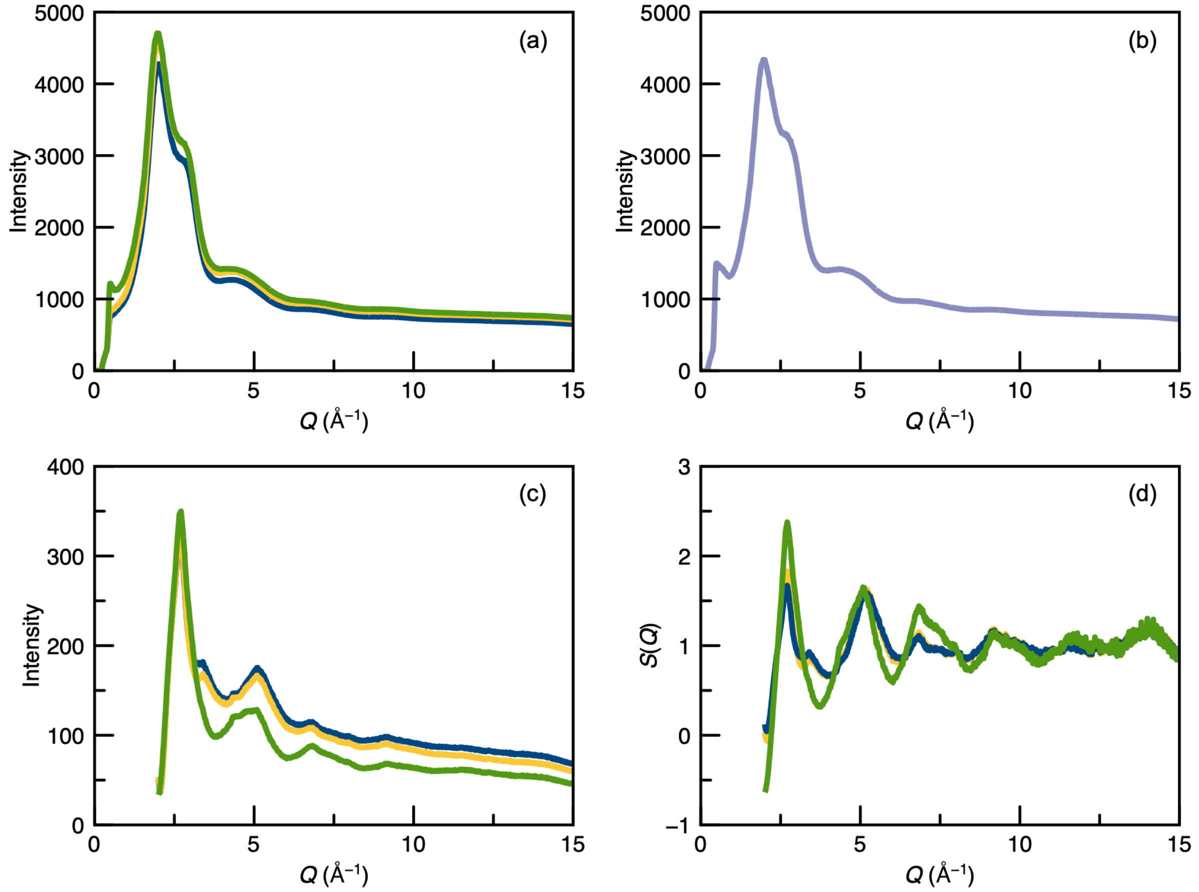

Figure S2 (a) Total scattering for water (blue), aqueous ammonium acetate (yellow) and aqueous DNA (green), each sealed in Kapton capillaries. **(b)** Total scattering from DNA_2-$[Ag_{16}Cl_2]^{8+}$ in solution. **(c)** Background corrected total scattering for DNA_2-$[Ag_{16}Cl_2]^{8+}$ in solution after subtraction of water (blue), aqueous ammonium acetate (yellow) and aqueous DNA (green). The solvent scattering dominated the signal, contributing around 95% of the total signal. **(d)** The corresponding structure factors for the data in **(c)**. The structure factors did not exhibit any Bragg peaks, as expected from small clusters in solution. Instead, broad regions of diffuse scattering are observed.

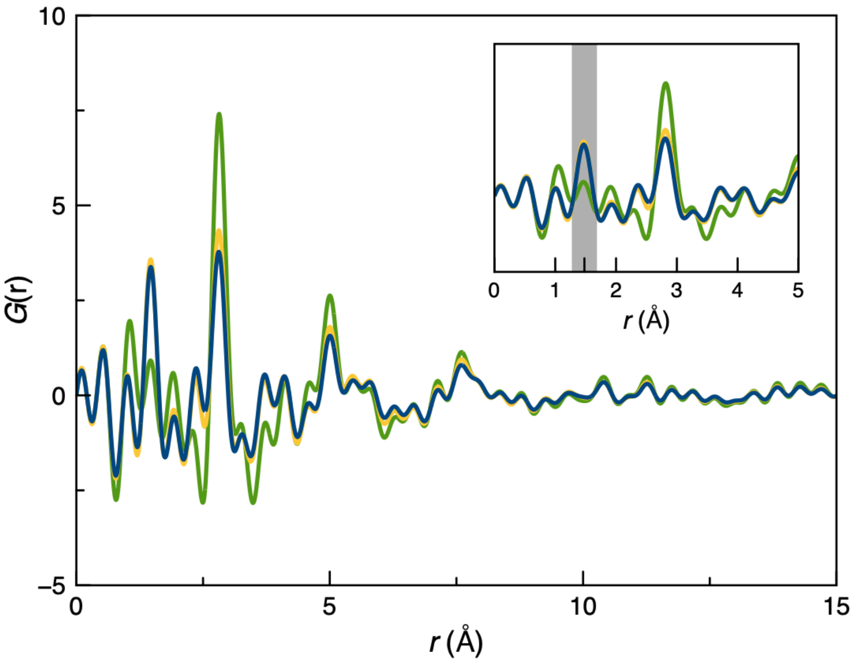

Figure S3 PDFs obtained from the DNA_2-$[Ag_{16}Cl_2]^{8+}$ sample after background subtraction with water (blue), aqueous ammonium acetate (yellow) and aqueous DNA (green). Inset shows the low-*r* region, and the grey region highlights the main contribution from the DNA (C–C/P–O bonds). The intensity of this peak is reduced almost to the baseline when aqueous DNA is used as the background contribution. Therefore, we can be confident that the local structure of the DNA is subtracted as best as possible.

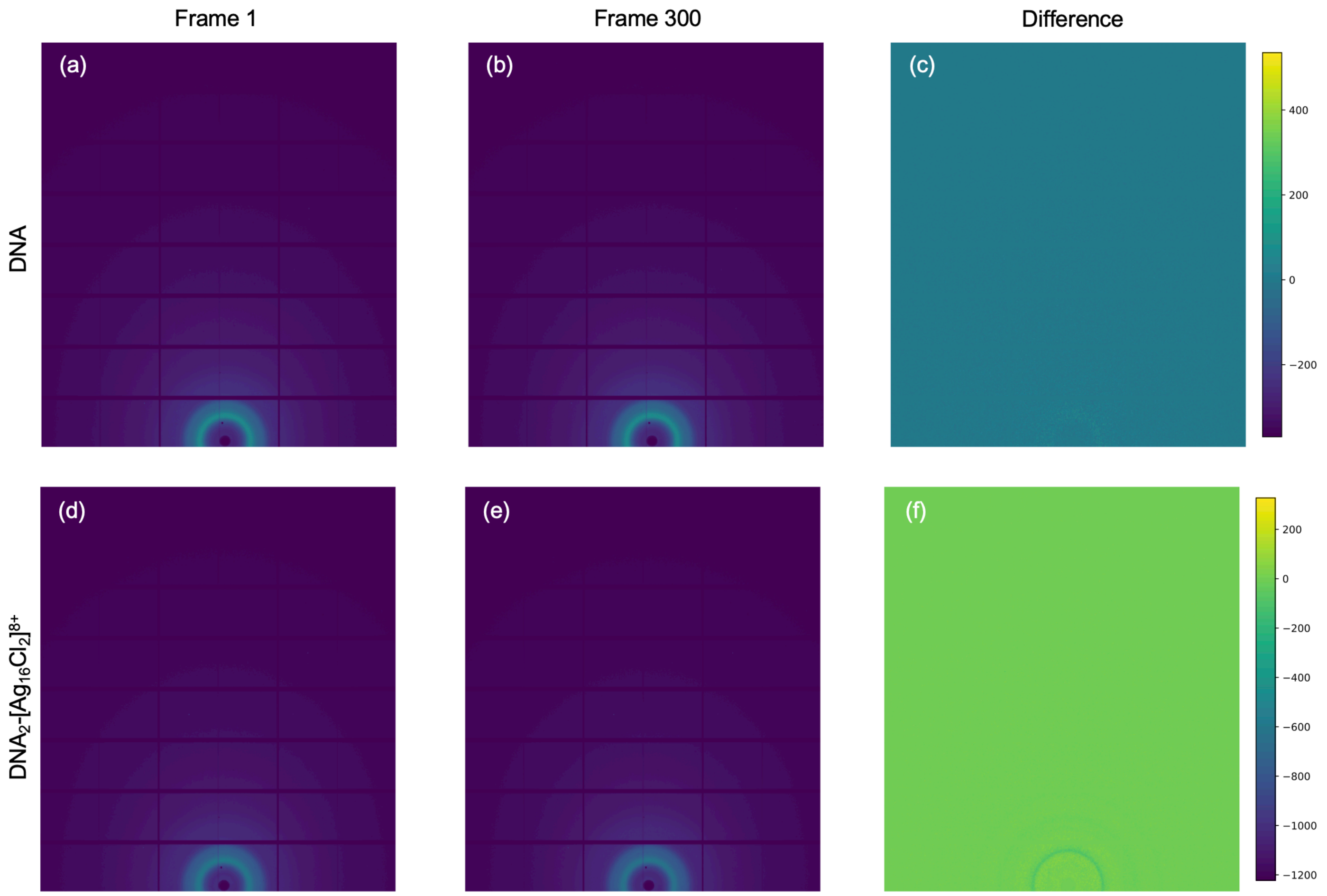

Figure S4 Given the potential instability of the DNA strands under X-ray irradiation, the first and last frame of the five minute measurements were assessed for DNA **(a to c)** and DNA_2-$[Ag_{16}Cl_2]^{8+}$ **(d to f)**. Subtraction of the raw detector images shows that almost negligible changes occur for the DNA and DNA_2-$[Ag_{16}Cl_2]^{8+}$ over the measurement.

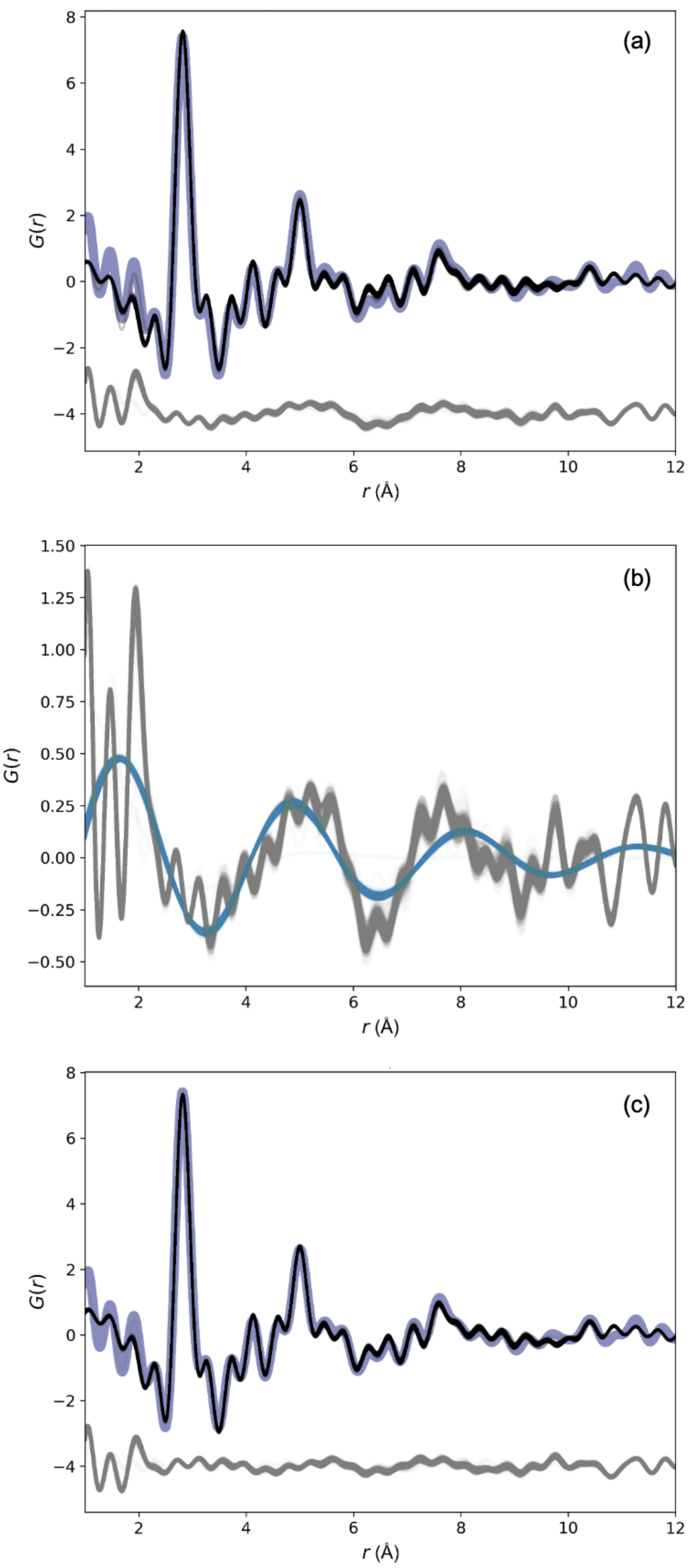

Figure S5 Results from the final refinement strategy. In each figure, the results from all 500 refinements are shown. **(a)** Experimental PDF (purple), refined PDF (model: $Ag_{16}Cl_2$) (black) and difference curve (grey). **(b)** Difference curves (grey) and refined empirical "wave" model (blue). **(c)** Experimental PDF (purple), refined PDF (model: $Ag_{16}Cl_2$ & empirical "wave") (black) and difference curve (grey).

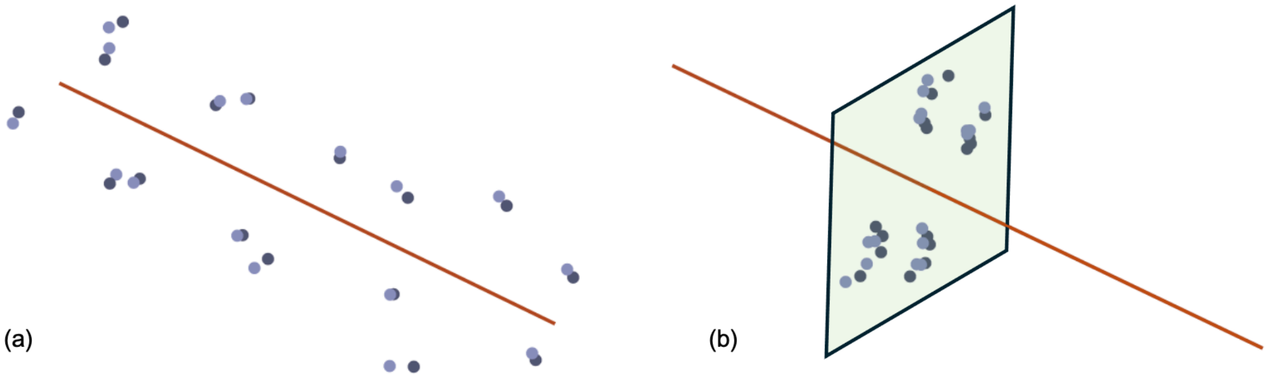

Figure S6 (a) Ag_{16} coordinates pre- (dark purple) and post- (light purple) refinement. The green line represents the principal axis of the points in space. **(b)** The atomic coordinates are then projected into a plane perpendicular to the principal axis. **Figure 4c** in the main text is obtained by viewing along the direction of the principal axis. The angle of rotation for each atom is determined about the principal axis.

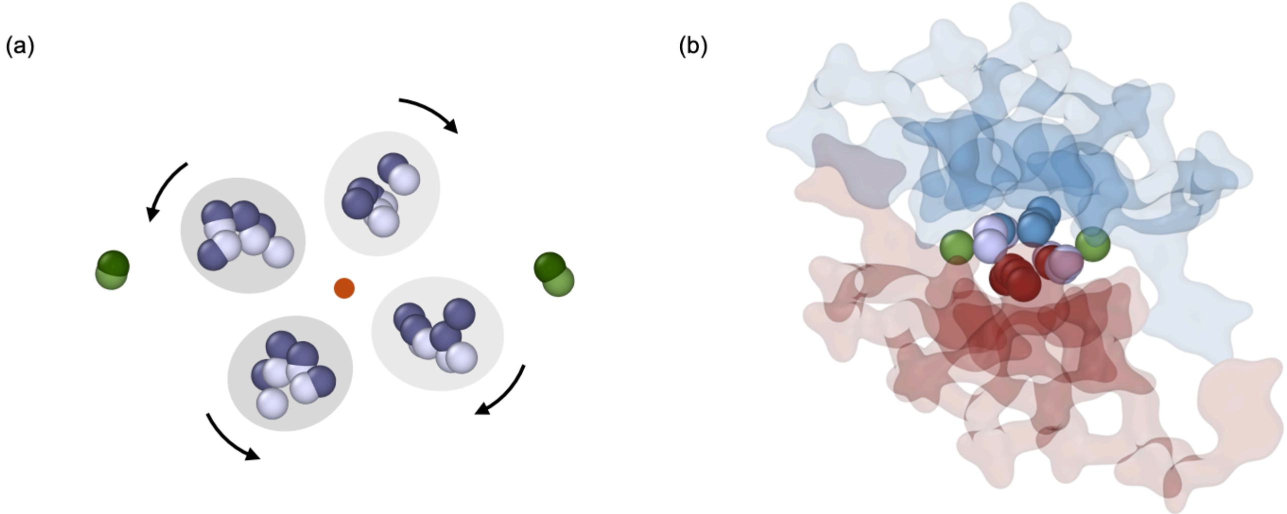

Figure S7 (a) Rotation analysis figure reproduced from the main text in **Figure 4c**. The original crystal (dark) and refined average (light) structures of the $Ag_{16}Cl_2$ core (Ag purple and Cl^- green). The core is viewed along the principal axis (orange dot). Groups of atoms displaying clockwise and counterclockwise rotations are highlighted in light and dark grey, respectively. **(b)** The original crystal structure of the $Ag_{16}Cl_2$ core is viewed in the same orientation as **(a)**, with the DNA oligomers shown as a semi-transparent surface mesh. The two separate oligomers are coloured in red and blue. In this representation, Ag coordinated solely by the blue oligomer are coloured blue, those coordinated solely by the red oligomer in red and those coordinated by both in purple. By comparing **(a)** and **(b)**, it can be seen that seven out of eight of the Ag involved in either the clockwise or counterclockwise rotation are either solely or partially coordinated by the same strand of DNA. For the clockwise rotation, this is the DNA strand in blue and the red strand for the counterclockwise rotation. For both directions, the opposite strand of DNA coordinates the remaining Ag.

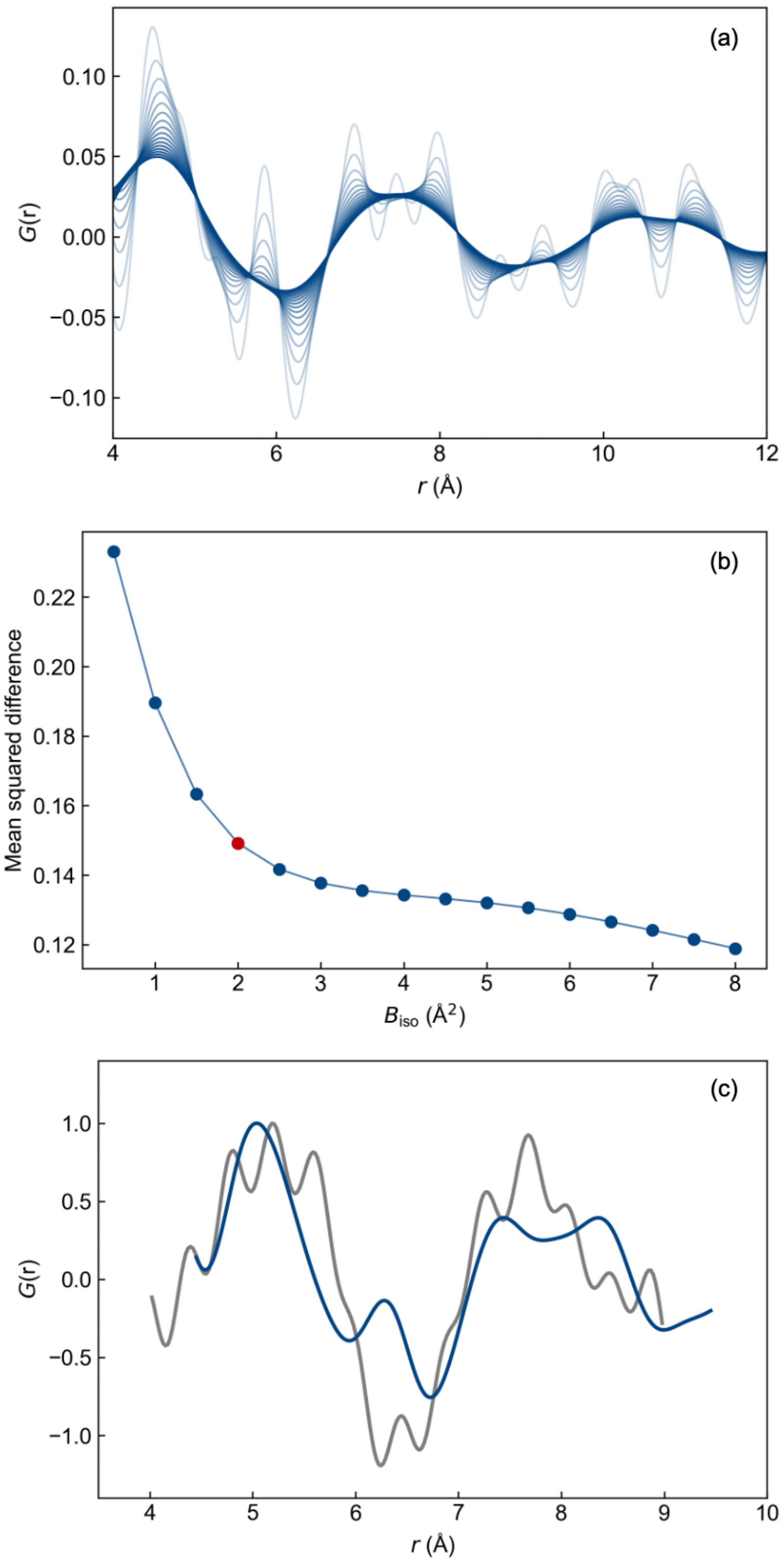

Figure S8 (a) PDFs calculated from DNA (crystal), with B_{iso} values between 0.5 (light blue) to 8.0 (dark blue) Å^2. **(b)** Mean squared difference between the refined calculated DNA (crystal) PDFs in **(a)** and the experimentally derived DNA (cluster) PDF. For each PDF in **(a)**, an *x*-shift to align the two broad regions in the PDF and a scale factor were refined, and the mean square difference (MSD) was then computed. The *x*-shift (*ca.* 0.44 Å) was necessary to align the features in the PDF, which we interpreted as a swelling effect in the conformation of the DNA in solution compared to the crystalline state, causing it to expand due to the excess solvent present. It was noted that the MSD monotonically decreased as B_{iso} increased and continued to do so for values of B_{iso} up to 50 Å^2. However, the reduction in MSD became increasingly marginal. Therefore, we determined the optimum B_{iso} value as a trade-off between minimising the MSD whilst keeping the value of B_{iso} physically meaningful. This was achieved by determining the "elbow" point in **(b)**, defined as the point of maximum curvature – this was determined to be 2.0 Å^2. **(c)** Comparison between the refined (*i.e.* *x*-shifted and *y*-scaled, to account for the swelling and intensity differences, respectively) calculated DNA (crystal) PDF (blue) and the DNA (cluster) experimental PDF (grey).

Appendix A

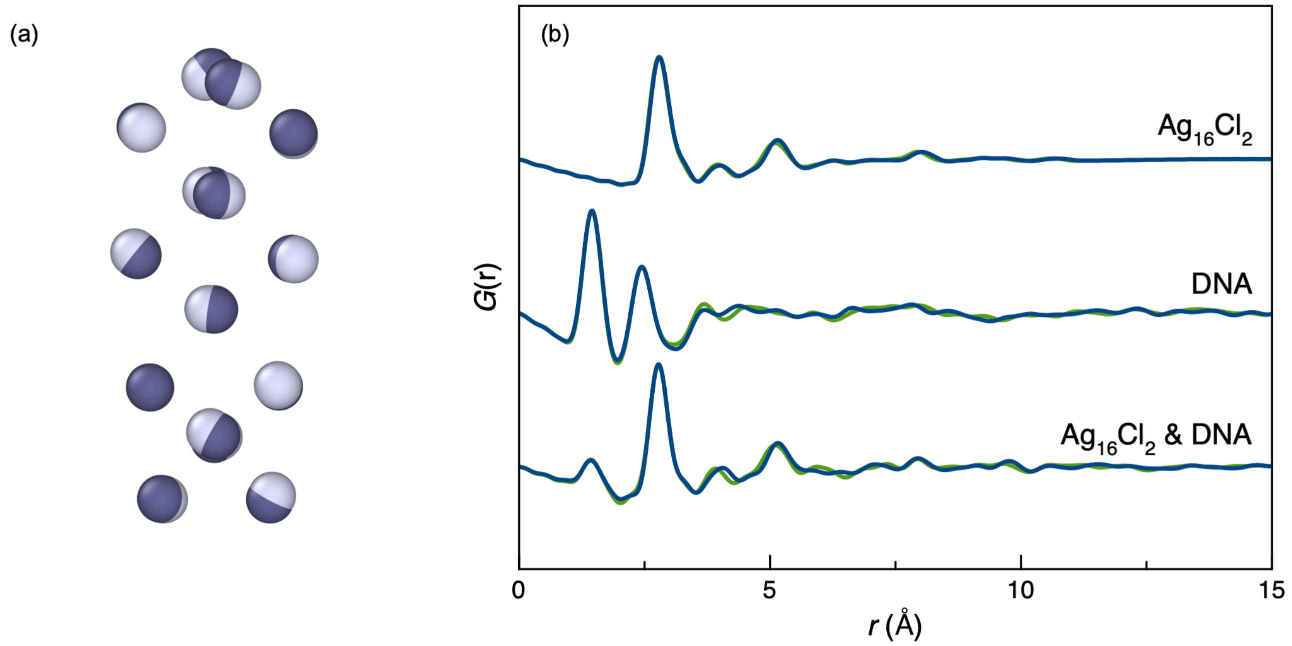

Figure S9 (a) Overlay of the Ag_{16} cores extracted from the reported single crystal structures with PDB codes *6jr4* (dark purple) and *7xlw* (light purple).[2] The mean displacement between Ag in the original and mutated form is 0.139 Å. The original, non-mutated structure *6jr4* was used for analysis in the main text.[3] **(b)** Comparison between the PDFs calculated from $Ag_{16}Cl_2$, DNA, and $Ag_{16}Cl_2$ & DNA units extracted from *6jr4* (green) and *7xlw* (dark blue). We note that the PDFs from the $Ag_{16}Cl_2$ core, which we are most sensitive to in our measurements, are almost identical.

References

(1) Bogh, S. A.; Carro-Temboury, M. R.; Cerretani, C.; Swasey, S. M.; Copp, S. M.; Gwinn, E. G.; Vosch, T. Unusually Large Stokes Shift for a Near-Infrared Emitting DNA-Stabilized Silver Nanocluster. *Methods and Applications in Fluorescence* **2018**, *6* (2), 024004. DOI: 10.1088/2050-6120/aaa8bc

(2) Cerretani, C.; Liisberg, M. B.; Rück, V.; Kondo, J.; Vosch, T. The Effect of Inosine on the Spectroscopic Properties and Crystal Structure of a NIR-Emitting DNA-Stabilized Silver Nanocluster. *Nanoscale Advances* **2022**, *4* (15), 3212–3217. DOI: 10.1039/D2NA00325B

(3) Cerretani, C.; Kanazawa, H.; Vosch, T.; Kondo, J. Crystal Structure of a NIR-Emitting DNA-Stabilized Ag_{16} Nanocluster. *Angewandte Chemie International Edition* **2019**, *58* (48), 17153–17157. DOI: 10.1002/anie.201906766

(4) Juhás, P.; Davis, T.; Farrow, C. L.; Billinge, S. J. L. PDFgetX3: A Rapid and Highly Automatable Program for Processing Powder Diffraction Data into Total Scattering Pair Distribution Functions. *Journal of Applied Crystallography* **2013**, *46* (2), 560–566. DOI: 10.1107/S0021889813005190

(5) Juhás, P.; Farrow, C. L.; Yang, X.; Knox, K. R.; Billinge, S. J. L. Complex Modelling: A Strategy and Software Program for Combining Multiple Information Sources to Solve Ill Posed Structure and Nanostructure Inverse Problems. *Acta Crystallography* **2015**, *71* (6), 562–568. DOI: 10.1107/S2053273315014473

(6) Gonzàlez-Rosell, A.; Malola, S.; Guha, R.; Arevalos, N. R.; Matus, M. F.; Goulet, M. E.; Haapaniemi, E.; Katz, B. B.; Vosch, T.; Kondo, J.; Häkkinen, H.; Copp, S. M. Chloride Ligands on DNA-Stabilized Silver Nanoclusters. *Journal of the American Chemical Society* **2023**, *145* (19), 10721–10729. DOI: 10.1021/jacs.3c01366

(7) Zobel, M.; Neder, R. B.; Kimber, S. A. J. Universal Solvent Restructuring Induced by Colloidal Nanoparticles. *Science* **2015**, *347* (6219), 292–294. DOI: 10.1126/science.1261412.

(8) Pedregosa, F.; Varoquaux, G.; Gramfort, A.; Michel, V.; Thirion, B.; Grisel, O.; Blondel, M.; Prettenhofer, P.; Weiss, R.; Dubourg, V.; Vanderplas, J.; Passos, A.; Cournapeau, D. Scikit-Learn: Machine Learning in Python. *J. Mach. Learn Res.* **2011**, *12,* 2825–2830.

(9) Mortensen, J. J.; Larsen, A. H.; Kuisma, M.; Ivanov, A. V.; Taghizadeh, A.; Peterson, A.; Haldar, A.; Dohn, A. O.; Schäfer, C.; Jónsson, E. Ö.; Hermes, E. D.; Nilsson, F. A.; Kastlunger, G.; Levi, G.; Jónsson, H.; Häkkinen, H.; Fojt, J.; Kangsabanik, J.; Sødequist, J.; Lehtomäki, J.; Heske, J.; Enkovaara, J.; Winther, K. T.; Dulak, M.; Melander, M. M.; Ovesen, M.; Louhivuori, M.; Walter, M.; Gjerding, M.; Lopez-Acevedo, O.; Erhart, P.; Warmbier, R.; Würdemann, R.; Kaappa, S.; Latini, S.; Boland, T. M.; Bligaard, T.; Skovhus, T.; Susi, T.; Maxson, T.; Rossi, T.; Chen, X.; Schmerwitz, Y. L. A.; Schiøtz, J.; Olsen, T.; Jacobsen, K. W.; Thygesen, K. S. GPAW: An Open Python Package for Electronic Structure Calculations. *Journal of Chemical Physics* **2024**, *160* (9), 092503. DOI: 10.1063/5.0182685

(10) Larsen, A. H.; Mortensen, J. J.; Blomqvist, J.; Castelli, I. E.; Christensen, R.; Dułak, M.; Friis, J.; Groves, M. N.; Hammer, B.; Hargus, C.; Hermes, E. D.; Jennings, P. C.; Jensen, P. B.; Kermode, J.; Kitchin, J. R.; Kolsbjerg, E. L.; Kubal, J.; Kaasbjerg, K.; Lysgaard, S.; Maronsson, J. B.; Maxson, T.; Olsen, T.; Pastewka, L.; Peterson, A.; Rostgaard, C.; Schiøtz, J.; Schütt, O.; Strange, M.; Thygesen, K. S.; Vegge, T.; Vilhelmsen, L.; Walter, M.; Zeng, Z.; Jacobsen, K. W. The Atomic Simulation Environment—a Python Library for Working with Atoms. *Journal of Physics: Condensed Matter* **2017**, *29* (27), 273002. DOI: 10.1088/1361-648X/aa680e

(11) Lehtola, S.; Steigemann, C.; Oliveira, M. J. T.; Marques, M. A. L. Recent Developments in Libxc — A Comprehensive Library of Functionals for Density Functional Theory. *SoftwareX* **2018**, *7*, 1–5. DOI: 10.1016/j.softx.2017.11.002

(12) Malola, S.; Matus, M. F.; Häkkinen, H. Theoretical Analysis of the Electronic Structure and Optical Properties of DNA-Stabilized Silver Cluster $Ag_{16}Cl_2$ in Aqueous Solvent. *Journal of Physical Chemistry C* **2023**, *127* (33), 16553–16559. DOI: 10.1021/acs.jpcc.3c04103

(13) Held, A.; Walter, M. Simplified Continuum Solvent Model with a Smooth Cavity Based on Volumetric Data. *Journal of Chemical Physics* **2014**, *141* (17), 174108. DOI: 10.1063/1.4900838.
